# Weight loss in individuals with metabolic syndrome given DASH diet counseling when provided a low sodium vegetable juice: a randomized controlled trial

**DOI:** 10.1186/1475-2891-9-8

**Published:** 2010-02-23

**Authors:** Sonia F Shenoy, Walker SC Poston, Rebecca S Reeves, Alexandra G Kazaks, Roberta R Holt, Carl L Keen, Hsin Ju Chen, C Keith Haddock, Barbara L Winters, Chor San H Khoo, John P Foreyt

**Affiliations:** 1 Department of Nutrition, University of California, Davis, USA; 2 Institute for Biobehavioral Health Research, National Development and Research Institutes (NDRI), Leawood, Kansas, USA; 3 Department of Medicine, Baylor College of Medicine, Houston, Texas, USA; 4 Department of Nutrition and Exercise Science, Bastyr University, Kenmore, Washington, USA; 5 Department of Internal Medicine, University of California, Davis, USA; 6 Campbell Soup Company, Camden, New Jersey, USA

## Abstract

**Background:**

Metabolic syndrome, a constellation of metabolic risk factors for type 2 diabetes and cardiovascular disease, is one of the fastest growing disease entities in the world. Weight loss is thought to be a key to improving all aspects of metabolic syndrome. Research studies have suggested benefits from diets rich in vegetables and fruits in helping individuals reach and achieve healthy weights.

**Objective:**

To evaluate the effects of a ready to serve vegetable juice as part of a calorie-appropriate Dietary Approaches to Stop Hypertension (DASH) diet in an ethnically diverse population of people with Metabolic Syndrome on weight loss and their ability to meet vegetable intake recommendations, and on their clinical characteristics of metabolic syndrome (waist circumference, triglycerides, HDL, fasting blood glucose and blood pressure).

A secondary goal was to examine the impact of the vegetable juice on associated parameters, including leptin, vascular adhesion markers, and markers of the oxidative defense system and of oxidative stress.

**Methods:**

A prospective 12 week, 3 group (0, 8, or 16 fluid ounces of low sodium vegetable juice) parallel arm randomized controlled trial. Participants were requested to limit their calorie intake to 1600 kcals for women and 1800 kcals for men and were educated on the DASH diet. A total of 81 (22 men & 59 women) participants with Metabolic Syndrome were enrolled into the study. Dietary nutrient and vegetable intake, weight, height, leptin, metabolic syndrome clinical characteristics and related markers of endothelial and cardiovascular health were measured at baseline, 6-, and 12-weeks.

**Results:**

There were significant group by time interactions when aggregating both groups consuming vegetable juice (8 or 16 fluid ounces daily). Those consuming juice lost more weight, consumed more Vitamin C, potassium, and dietary vegetables than individuals who were in the group that only received diet counseling (p < 0.05).

**Conclusion:**

The incorporation of vegetable juice into the daily diet can be a simple and effective way to increase the number of daily vegetable servings. Data from this study also suggest the potential of using a low sodium vegetable juice in conjunction with a calorie restricted diet to aid in weight loss in overweight individuals with metabolic syndrome.

## Background

Metabolic syndrome, a constellation of metabolic risk factors for type 2 diabetes and cardiovascular disease, is one of the fastest growing disease entities in the world [1,2]; as an example in the United States it is thought to affect over 30% of adults [3]. Weight loss is thought to be a key to improving all aspects of metabolic syndrome [4]. Research studies have suggested a number of benefits of diets rich in vegetables and fruits in helping individuals reach and achieve healthy weights [5]. Vegetables and fruits, which are typically low in calories, can provide an abundance of essential nutrients and health promoting phytochemicals [6]. Clinical science and public health data underscore the potential health benefits that could be realized if vegetable intakes matched current dietary recommendations [7]. Regrettably, adopting and maintaining a healthy lifestyle, including a diet rich in vegetables, fruits, lean meats and low fat dairy products, seems to be problematic for many individuals, even when they are aware of its benefits [8]. For example, McGee et al. reported that focus group participants from the Lower Mississippi Delta with chronic disease risk factors resisted adopting a healthy diet, when it meant giving up traditional or culture-related dietary habits [9]. Data show that older ethnic minorities do not meet the minimum recommendations for vegetables and fruits [10]. Preparation time [9], price [11], taste [12], and lack of convenience [13] are among barriers that have been reported to contribute to the low consumption of vegetables and fruits.

Research on the favorable effects of vegetables and fruits and their phytochemicals is expanding rapidly with data showing positive impacts of many plant foods on risk factors for chronic diseases [14]. Specific to metabolic syndrome, diets high in vegetables have been reported to have beneficial effects with respect to fasting blood glucose, dyslipidemia, and hypertension [15-18]. While it could be argued that the above positive effects of high vegetable diets may simply reflect the adoption of "healthy diets," there is increasing evidence that some of the reported positive effects may be linked to specific phytochemicals. For example, it has been reported that carotenoids can inhibit damage and thickening of the arterial wall, possibly due to their ability to lower the production of select inflammatory cytokines [19]. Similarly, data with an inverse association of plasma lycopene levels and intima thickening, one index of cardiovascular disease [20] has been reported. Two different double-blind, placebo-controlled trials, showed a tomato extract significantly reduced systolic and diastolic blood pressure; one study had patients with grade 1 hypertension [21], and another with moderate hypertension despite anti-hyptertensive medication at enrollment [22]. In another study, beneficial results on platelet function were reported in healthy volunteers after drinking a tomato extract [23,24]. Research is increasing not only on the individual phytochemicals, but also on their potential synergistic health benefits [25].

The primary goal of the present research was to examine the effects of consuming 8 or 16 fluid ounces of low sodium vegetable juice as part of a calorie-appropriate Dietary Approaches to Stop Hypertension (DASH) diet in an ethnically diverse population on the ability of vegetable juice to help subjects lose weight, meet their recommended vegetable intake, and on clinical characteristics of metabolic syndrome (waist circumference, triglycerides, HDL, fasting blood glucose and blood pressure). A secondary goal was to examine the impact of the incorporation of vegetable juice in the diet on associated parameters, including leptin, vascular adhesion markers, and markers of the oxidative defense system and oxidative stress.

## Methods

### Study Population and Setting

Adult men and women, ages 35-65, were recruited from the Houston, TX community using advertisements placed on several key radio stations, in local neighborhood and business newspapers, free newspapers containing advertising, and the Baylor College of Medicine health newsletter. Two hundred and fifty-three individuals were screened in the clinic and 81 (59 women and 22 men) met inclusionary criteria and were randomized into the study. Those who responded to our advertisements and met the clinical criteria tended to be minorities. Specifically, the participants included: 57% African-American, 23% Mexican American, 17% Caucasian, and 4% other.

Participants enrolled into the study met the criteria set by the National Cholesterol Education Program (NCEP) Adult Treatment Panel (ATP) Panel III for metabolic syndrome defined as meeting at least three out of the five following parameters: 1) waist circumference for men > 40 in, for women > 35 in; 2) triglycerides > 150 mg/dl; 3) systolic blood pressure > 130 mm Hg or diastolic blood pressure > 85 mm Hg; 4) fasting blood glucose > 100 mg/dl; 5) HDL-cholesterol < 40 mg/dl for men and < 50 mg/dl for females. Body mass indices (BMIs) of the eligible participants could range from 30-50 kg/m^2^.

Participants were excluded from the study for the following reasons: use of anxiolytics or antidepressive medication, hormone replacement therapy, reported alcohol consumption in excess of 1 fluid ounce/day, diabetes controlled with insulin, hyper- or hypothyrodism, inflammatory disorders, treatment with corticosteroids and anti-inflammatory drugs, routine use of aspirin and other NSAIDs, or a history of a major cardiovascular event. The following clinical parameters were exclusionary: abnormal complete blood cell count defined as low/high WBCs (less than 4.0 K/mm^3 ^or greater than 11.0 K/mm^3^), hemoglobin (less than 11.5 or greater than 17.0 g/dL), platelets (less than 130 K/mm^3 ^or greater than 450 K/mm^3^), or a Beck Depression Inventory^® ^(BDI) scale score of 21 or above (Pearson Education, Inc., San Antonio, Texas). With the exception of basic multivitamin/mineral supplements, subjects were instructed to refrain from using dietary supplements, including herbs and omega-3 fatty acids during the study period. Participants were instructed to refrain from using nonsteroidal and anti-inflammatory medications for the week prior to a clinic visit. All subjects provided written informed consent at the time of screening, and the Institutional Review Board at Baylor College of Medicine approved this study.

### Study Design

Eligible subjects were randomized into one of three groups: (1) 8 fluid ounces of low sodium vegetable juice/day; (2) 16 fluid ounces of low sodium vegetable juice/day; or (3) no vegetable juice/day, for a 12-week period. Clinic visits were at baseline (week 0), week 6 and week 12 of the study. Subjects were instructed to follow a low carotenoid diet for the week prior to the baseline visit and a low flavonoid diet 24 hours prior to all visits. Previous studies suggest that these dietary phytochemicals can have a positive impact on vascular function [26,27]. Three day diet records were collected prior to the low carotenoid washout diet at baseline and prior to the 24 hour low flavonoid diet at the 6 and 12 week visits.

All participants were asked to follow a calorie-controlled DASH diet plan. Men were asked to follow an 1800 kcal diet and women a 1600 kcal diet. DASH is an eating pattern recommended by the 2005 Department of Health and Human Services Dietary Guidelines for Americans as a model of healthy eating for the majority of individuals in the population [28]. The DASH diet emphasizes vegetables, fruits, whole grains, lean meats and low fat dairy foods, and is rich in magnesium, potassium, calcium and fiber [29]. At the baseline visit following randomization to one of three groups, all participants spent about 45 minutes with a dietitian learning the basics of the DASH diet. Dietitians emphasized the following points of the nutrition education material to participants:

1. Key aspects of the DASH eating plan placing emphasis on vegetables and fruits.

2. Appropriate serving sizes of foods.

3. Realistic personal goals and meal plans.

4. Tips to make healthy eating easier.

5. Checklist to track their individual progress towards meeting the DASH goals.

A notebook containing relevant DASH nutrition education material was provided to participants. At the 6 week visit, a brief follow-up session was again conducted with dietitians who asked about progress in following the diet.

Participants randomly assigned to the beverage groups were supplied with the low sodium vegetable juice for each 6 week period. The juice was packaged in 46-ounce bottles with a plain black and white label. The same manufacturing lot was used for all subjects for the 12-week study period. A clear plastic glass with an 8 fluid ounce marker was provided for ease of juice measurement. Eight fluid ounces of the low sodium vegetable juice (V8^®^; Campbell Soup Company, Camden NJ) provided 50 calories, 0 g of total fat and cholesterol, 140 mg of sodium, 820 mg of potassium, 2 g of protein, 20 mg lycopene, and 10 g of total carbohydrate of which 2 g were dietary fiber. The juice provided 40% of the Daily Value of Vitamin A from naturally occurring beta-carotene in the vegetables (1000 IUs = 300 micrograms RAEs (Retinol Activity Equivalents)), 120% of Vitamin C, and 2% of calcium and iron.

### Data Collection and Measures

General health, medication use and lifestyle characteristics were assessed at baseline. At weeks 6 and 12, subjects who consumed 8 or 16 fluid ounces of juice/day completed an 8-item Beverage Consumption Questionnaire that included questions about the perceived taste and health benefits of the beverage. Daily beverage consumption was reported on checklists to measure adherence to their allotted juice group protocol. Similar to the literature, subjects were deemed highly adherent to the protocol if they consumed their allotted amount of juice at least 85% of the study days (72 of the 84 days of the trial) [30,31]. Three-day food records were collected from 2 weekdays and 1 weekend day before study visits at baseline, week 6 and week 12. The food records were reviewed by a registered dietitian when they were submitted and then were sent to the University of California, Davis where a registered dietitian supervised duplicate data entry and analysis using Food Processor software (Version 10.2.0, ESHA research, Inc., Salem, OR). Vegetable servings were quantified according to MyPyramid cup servings [32].

Clinical measurements included blood pressure, weight, height, and waist circumference. Blood pressure measurements were the average of 2 measurements and were taken using an automated system (Dinamap Pro 100 by GE, Criticon, Tampa, FL.) after the subjects were seated for 5 minutes. For weight and height measurements, subjects were fully dressed, with the exception that their shoes were removed. Height was recorded on their first visit using a wall-mounted stadiometer (Accustat Genentech, San Francisco, CA). Weight was recorded every visit using an electronic scale (Tanita, BWB--800. Tokyo, Japan.). Body mass index (kg/m^2^) was calculated as weight (kg) divided by height squared (m^2^).

At the screening visit, blood samples were drawn for the comprehensive metabolic panel (chemistry, lipid, fasting blood glucose, liver function and complete blood count) and analyzed at the Clinical Pathology Laboratory in Austin, TX. At baseline, 6 and 12 weeks, blood samples for lipids, high sensitivity C-reactive protein (hsCRP), glycated hemoglobin (HgA1c) and insulin were analyzed at the Atherosclerosis Clinical Research Laboratory, a core laboratory in the Department of Medicine at Baylor College of Medicine. Plasma was collected for the measurement of adhesion markers, leptin, and for plasma indicators of oxidant defense (total reactive antioxidant potential (TRAP)) and oxidative damage (thiobarbituric acid reactive substances (TBARS)). TRAP and TBARS were analyzed as previously described [33].

Leptin, and vascular adhesion markers (soluble-Intercellular Adhesion Molecule-1, soluble-Vascular Cell Adhesion Molecule-1, soluble P-Selectin, soluble E-Selectin), and soluble CD40 ligand were measured using a commercially available enzyme-linked immunosorbent assay (ELISA) kits (leptin, adhesion markers: R&D Systems, Minneapolis, MN, sCD40L: Bender MedSystems, Burlingame, CA) according to the manufacturer's instructions.

### Statistical Approach

Descriptive data (means, standard deviations) are provided for study outcomes stratified by the three study conditions. For weight change, descriptive data also is presented after applying imputational methods (described below) for modeling missing data.

#### Changes in Body Weight

Three different unadjusted statistical models were created to examine the impact of the three study conditions on body weight. The first model examined weight loss among completers of the three treatment conditions. Next, two models which imputed treatment outcome data for participants who dropped out of the study were developed. The first imputational model was based on the Last Observation Carried Forward (LOCF) [34,35] method. The second imputation model used a conservative Intention-to-Treat (ITT) method where missing values are imputed based on average weight gain after dropout of 0.30 kg/month (or 0.075 kg/week) after study withdrawal, an approach that has been used successfully in other large clinical trials [35,36] and is even more conservative because it assumes that weight regain can exceed baseline weight. For all three models, General Linear Models for repeated measures were developed where the between-subjects factor was group assignment and the within-subjects factor was body weight at baseline, 6 weeks, and 12 weeks. Data were examined based on a comparison of the results from the three methods for modeling missing data. A multivariate approach was used to test a group by time interaction in each model based on the Wilks Lambda test of significance. The multivariate test was conducted on difference scores, and therefore the assumptions underlying the multivariate test concern these difference scores. Difference scores (change from baseline) for weight outcomes are presented to aid interpretability. LSD and the more conservative Tukey HSD post-hoc comparisons were used for any statistically significant unadjusted model.

#### Changes in Leptin, Adhesion Markers, CD40L, Blood Pressure, and Food Record Data

General linear models with repeated measures were used to examine changes in leptin, adhesion markers, blood pressure, and food diary data for the three study conditions. All models are based on participants who completed the study given that imputational approaches for small samples are not well developed for these factors. Tukey post-hoc comparisons were used for any statistically significant unadjusted model.

#### Adjusted Aggregate Models

Given the attrition observed in the study, the resultant reduction in statistical power, and the fact that no differences were found between the 8 fluid ounce low sodium vegetable juice and 16 fluid ounce juice conditions on any outcome, the 8 and 16 fluid ounce juice groups were aggregated into a single group and the aggregated condition was compared to the group that did not consume the juice (control group). Thus, aggregate models were developed to compare any low sodium vegetable juice consumption to none. In addition, gender, education, and age were included as covariates in the adjusted models. These covariates were selected because of the relatively large differences in their distribution by group status even though the differences were not statistically significant, as well they were used because have been previously demonstrated to be related to weight loss outcomes [37-39].

Medication use was also examined to assess whether inclusion of a measure of medication use (i.e., number of medications used) enhanced the precision of outcomes models. This was implemented because outcome variables might be affected by prescription and OTC medication use, i.e., weight, leptin, systolic and diastolic blood pressure, and all lipid fractions. Any listed substance was excluded that was not clearly a medication (i.e., any nutritional supplement or vitamin). However, clear distinctions between prescription and over the counter (OTC) medications was not possible because some OTC drugs were prescribed (e.g., aspirin) and some participants did not list an actual medication, but instead a class of medications (e.g., "blood pressure medicine" or "allergy pills"). Thus, all OTC and prescription medication were grouped and counted as the total number of medications for each participant. The simplified models with juice consumption aggregated showed that that the addition of frequency of medication use did not substantially improve already significant models and did not change models that were previously not statistically significant.

## Results

**Figure 1:**
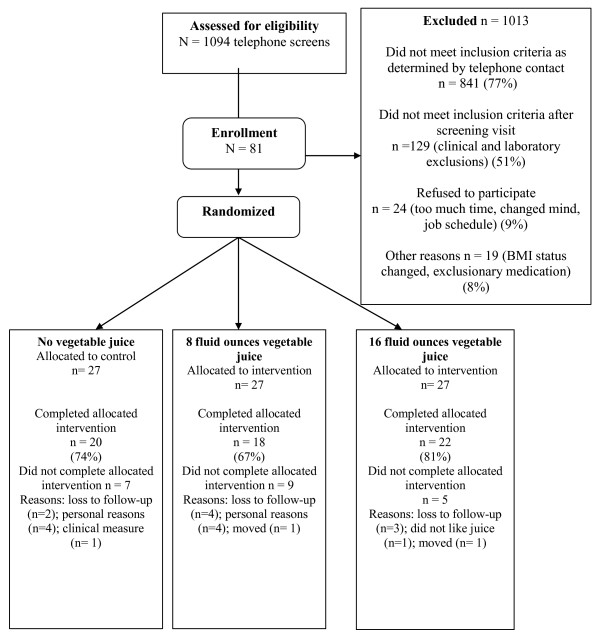
**Enrollment and Randomization**.

A total of 81 individuals participated in the study (27 in each study condition) (Figure 1). Baseline characteristics of the participants were similar among groups (Table 1). Overall retention was 74% and attrition was similar across groups. None of the baseline characteristics or treatment group status were associated with dropping out of the study.

**Table 1:** Participant Characteristics by Group Assignment (Mean or %, SD)

	Group Assignment			
**Demographic and Clinical Characteristics**	**No Vegetable Juice**	**8 fluid ounces Vegetable Juice** **Per Day**	**16 fluid ounces Vegetable Juice** **Per Day**	**All Participants**

Female* (%)	74.1	85.2	59.3	72.8
Ethnicity (%)				
-White	19.2	15.4	14.8	16.5
-African-American	50.0	57.7	63.0	57.0
-Mexican-American	30.8	23.1	14.8	22.8
-Other	0.0	3.8	7.4	3.7
Education: High School or Less (%)	56.0	62.5	58.3	58.9
Current Smoker (%)	14.8	14.8	11.5	13.3
Age (Years)	50.1 (5.1)	51.2 (7.4)	48.0 (7.7)	49.8 (6.9)
Baseline Weight (kg)	104.2 (18.1)	100.2 (17.6)	112.4 (18.1)	105.6 (18.4
Baseline BMI	37.8 (4.6)	37.3 (5.3)	38.3 (4.9)	37.8 (4.9)
Baseline Waist Circumference (cm)	115.3 (13.3)	112.9 (10.5)	119.1 (15.2)	115.8 (13.3)
Baseline Systolic Blood Pressure (mmHg)	132.8 (20.9)	134.5 (20.6)	124.4 (15.3)	130.6 (19.4)
Baseline Diastolic Blood Pressure (mmHg)	84.8 (11.8)	83.8 (11.5)	80.5 (9.9)	83.0 (11.1)
Baseline Medication Use (#)	1.7 (2.0)	1.8 (2.2)	1.0 (1.6)	1.5 (2.0)

Baseline Metabolic Syndrome Criteria (% with criteria)				

Waist Circumference (≥ 88 cm for women or ≥ 102 cm for men; %)	100.0	100.0	100.0	100.0
Blood Pressure (Systolic ≥ 130 mmHg or Diastolic ≥ 85 mmHg; %)	63.0	51.9	40.7	51.9
Triglycerides (≥ 150 mg/dl; %)	37.0	25.9	23.1	28.9
HDL (Men <40 mg/dl or Women <50 mg/dl; %)	96.0	80.8	87.5	88.0
Blood Glucose (≥ 100 mg/dl; %)	100.0	96.3	100.0	98.7

*χ^2 ^= 4.6, p = 0.10

We observed that 100% of subjects in the 8 fluid ounce/day group had high rates of adherence (i.e., beverage consumption on >85% of days in the study) over the 12 week trial, whereas only 53% of subjects in the 16 fluid ounce/day group had the same level of adherence.

### Weight Loss

All three models (i.e., completers, LOCF, and conservative ITT) demonstrated that participants in the two vegetable juice groups lost more weight, on average, than the group that did not drink the juice. However, there were no statistically significant group differences in weight loss over time (i.e., group by time interaction) (Table 2). When using adjusted, aggregated models, (vegetable juice vs. no vegetable juice) the group by time interaction tests for weight were statistically significant for completers (F = 4.3, p = 0.02; data not shown) and the LOCF and ITT models (F = 3.8, p = 0.03 for both; data not shown), indicating that participants who consumed one or more servings of vegetable juice experienced significantly more weight loss than those who did not consume the juice.

**Table 2:** Weight Loss (kg; M, SD) Over Time by Group Status for Completers, LOCF, and Conservative ITT Models

	Treatment Group					
**Weight Loss Marker**	**No Vegetable Juice**		**8 fluid ounces Vegetable Juice**		**16 fluid ounces Vegetable Juice**	
	
	**6 Weeks**	**12 Weeks**	**6 Weeks**	**12 Weeks**	**6 Weeks**	**12 Weeks**

Completers (kg) N	19	20	18	18	23	22
Mean	0.26	-0.55	-1.36	-2.25	-0.97	-1.35
SD	1.11	2.77	1.83	2.72	2.13	2.72
LOCF (kg) N	27	27	27	27	27	27
Mean	0.19	-0.41	-0.90	-1.55	-0.82	-1.20
SD	0.93	2.38	1.62	2.43	1.99	2.52
ITT (kg) N	27	27	27	27	27	27
Mean	0.32	-0.17	-0.75	-1.20	-0.76	-0.93
SD	0.93	2.45	1.72	2.67	2.02	2.60

LOCF = Last Observation Carried Forward

ITT = Intent to Treat

### Leptin

Both unadjusted and adjusted statistical models were created to examine the impact of the three study conditions on leptin. Table 3 presents changes in leptin by group status over the 12-week trial. The unadjusted model for leptin was statistically significant between the groups over the 12 week study period (F = 3.4, p = 0.01). Similarly, in the adjusted model of aggregated vegetable juice groups, there was a significant group by time interaction for leptin (Table 3; F = 3.4, p = 0.04) that paralleled weight loss. Additionally, post-hoc paired t-tests comparing baseline levels to both 6- and 12-week only showed significant changes in those consuming 8 fluid ounces of vegetable juice (respectively, t = 2.578, p = 0.02; t = 3.767, p = 0.002).

**Table 3:** Means (SD) for Leptin

Leptin* (pg/ml)		Baseline	6-weeks	12-weeks
	No Vegetable Juice	2.93 × 10^4^ (1.99 × 10^4^)	3.07 × 10^4^ (2.30 × 10^4^)	3.57 × 10^4^ (3.09 × 10^4^)

	8 fluid ounces Vegetable Juice	4.30 × 10^4^ (1.81 × 10^4^)	3.51 × 10^4^ (1.33 × 10^4^)	3.16 × 10^4^ (1.12 × 10^4^)

	16 fluid ounces Vegetable Juice	3.87 × 10^4^ (2.99 × 10^4^)	4.47 × 10^4^ (2.85 × 10^4^)	4.27 × 10^4^ (2.97 × 10^4^)

*p-value = 0.01 for overall repeated measures model interaction among treatment and change from baseline to 12 weeks.

### Blood Pressure and Plasma Measurements

Systolic or diastolic blood pressure was not statistically significantly changed (data not presented) between groups over the 12 week study period. No significant differences were observed in the markers of oxidant defense or oxidative stress (TRAP and TBARS respectively) that were assessed in the study (data not presented). No significant differences were observed in any of the vascular adhesion markers, hsCRP, HgA1c, and sCD40L (data not shown).

### Food Records Data

Table 4 presents food record data of selected nutrients for completers stratified by treatment condition. Table 5 shows vegetables intake by cups, in accordance with the MyPyramid definition [32], and by study treatment group, first with counting the vegetable juice followed by without vegetable juice as part of the sum of vegetable intake. Unadjusted General Linear Models examining group by time interactions among completers for food records were first computed. As shown in Tables 4 and 5, groups consuming vegetable juice increased their intake of vitamin C (F = 6.5, p < 0.001), potassium (F = 3.9, p < 0.002), and vegetables (F = 4.3, p = 0.003) over time compared to those who did not consume juice.

**Table 4:** Food Record Data: Completers Juice Data Included

Nutrient	Measurement Point		
	
Mean (SD)	Baseline	6 Weeks	12 Weeks
Total Calories			

No Vegetable Juice	1898.5 (599.9)	1986.7 (687.6)	1865.9 (667.3)
8 fluid ounces Vegetable Juice	2015.9 (931.9)	1672.6 (1146.7)	1654.7 (571.6)
16 fluid ounces Vegetable Juice	2184.0 (703.6)	2030.1 (984.4)	1676.7 (429.7)

Percent of Calories from Fat			

No Vegetable Juice	37.5 (7.4)	31.7 (14.1)	33.8 (7.5)
8 fluid ounces Vegetable Juice	39.3 (7.8)	33.9 (16.2)	29.7 (9.5)
16 fluid ounces Vegetable Juice	35.8 (7.4)	27.9 (13.4)	32.4 (9.7)

Protein Intake (g)			

No Vegetable Juice	84.2 (29.1)	89.8 (36.4)	78.2 (30.3)
8 fluid ounces Vegetable Juice	79.6 (27.2)	74.3 (35.1)	63.5 (16.1)
16 fluid ounces Vegetable Juice	95.6 (36.6)	91.2 (37.1)	76.4 (19.2)

Carbohydrate Intake (g)			

No Vegetable Juice	209.9 (60.0)	244.0 (100.7)	234.3 (103.3)
8 fluid ounces Vegetable Juice	224.2 (106.4)	195.2 (64.5)	220.3 (73.5)
16 fluid ounces Vegetable Juice	256.2 (96.1)	219.3 (80.5)	205.4 (75.7)

Fat Intake (g)			

No Vegetable Juice	80.8 (37.4)	68.8 (37.0)	70.3 (30.5)
8 fluid ounces Vegetable Juice	90.6 (55.3)	54.5 (31.8)	58.3 (34.0)
16 fluid ounces Vegetable Juice	86.3 (32.8)	56.6 (24.2)	61.8 (29.0)

Vitamin A Carotenoid (RE)			

No Vegetable Juice	157.0 (191.4)	541.4 (826.6)	424.7 (544.1)
8 fluid ounces Vegetable Juice	514.6 (557.2)	601.3 (788.6)	491.6 (628.0)
16 fluid ounces Vegetable Juice	218.0 (264.7)	403.8 (479.1)	485.5 (528.1)

Vitamin C* (mg)			

No Vegetable Juice	65.9(77.9)	69.3(45.6)	97.8(76.5)
8 fluid ounces Vegetable Juice	88.8(64.5)	132.5(30.1)	146.1(50.2)
16 fluid ounces Vegetable Juice	63.5(44.1)	236.8(108.4)	240.7(91.5)

Sodium (mg)			

No Vegetable Juice	3589.7 (1372.2)	3386.2 (1213.2)	3775.2 (1572.8)
8 fluid ounces Vegetable Juice	3274.5 (1492.2)	2699.2 (1273.4)	2707.6 (1133.3)
16 fluid ounces Vegetable Juice	4283.0 (3843.3)	2697.6 (959.2)	2995.7 (1100.5)

Potassium* (mg)			

No Vegetable Juice	1784.3 (777.4)	1954.8 (892.7)	1695.0 (675.5)
8 fluid ounces Vegetable Juice	2004.1 (824.9)	2391.3 (645.8)	2496.0 (776.2)
16 fluid ounces Vegetable Juice	2057.1 (1029.0)	3170.0 (868.2)	3341.4 (822.8)

Fiber (g)			

No Vegetable Juice	17.5(8.1)	18.0(8.4)	19.3(9.4)
8 fluid ounces Vegetable Juice	20.6(12.2)	19.2(8.4)	16.9(5.3)
16 fluid ounces Vegetable Juice	16.8(8.6)	20.5(10.3)	18.6(7.3)

*p-value < 0.05 for overall repeated measures model interaction among treatment and change from baseline to 12 weeks.

**Table 5:** Vegetable Intake from the Food Records Including and Excluding V8 Consumption

Including Vegetable Juice			
	**Baseline**	**6 Weeks**	**12 Weeks**

MyPyramid Vegetables*	N = 54	N = 46	N = 53

No Vegetable Juice	1.2 (0.9)	1.7 (1.3)	1.7 (1.3)

8 fluid ounces Vegetable Juice	1.9 (1.1)	2.5 (1.0)	2.9 (1.5)

16 fluid ounces Vegetable Juice	1.3 (0.8)	3.0 (1.1)	3.4 (1.0)

**Excluding Vegetable Juice**			

MyPyramid Vegetables	N = 54	N = 46	N = 53

No Vegetable Juice	1.2 (0.9)	1.7 (1.3)	1.7 (1.3)

8 fluid ounces Vegetable Juice	1.9 (1.1)	1.5 (1.0)	2.0 (1.5)

16 fluid ounces Vegetable Juice	1.3 (0.8)	1.3 (0.9)	1.6 (0.9)

*p-value < 0.01 for overall repeated measures model interaction among treatment and change from baseline to 12 weeks.

Vitamin C post-hoc analyses revealed that intakes were significantly higher in the 16 fluid ounce/day group than in the 8 fluid ounces/day group (p = 0.002 and 0.005 for LSD and Tukey HSD, respectively). Vitamin C intakes were also higher in the 16 fluid ounce/day group versus the group not consuming any juice (p < 0.001 for both LSD and Tukey HSD). Using the LSD test, those who drank 8 fluid ounces/day of vegetable juice reported higher vitamin C intakes than those who did not drink the juice (p = 0.03). However, the difference between the 8 fluid ounces/day vegetable juice and no vegetable juice group was not significant using the Tukey HSD (p = 0.07).

With respect to potassium, post-hoc analyses showed those who consumed 16 fluid ounces/day vegetable juice reported higher intake relative to the 8 fluid ounces/day group (p = 0.001 and 0.004 for LSD and Tukey HSD, respectively). Those who consumed no vegetable juice had significantly lower intakes of potassium than those consuming 16 fluid ounces of juice (p < 0.001 for both LSD and Tukey HSD). The difference between the 8 fluid ounces/day vegetable juice and the no juice group was not statistically significant for either post-hoc test (p = 0.06 and 0.13 for LSD and Tukey HSD, respectively).

Post-hoc analyses of vegetable intake using the MyPyramid vegetable definition illustrated those who did not incorporate the vegetable juice into their diet reported significantly less vegetable intake than those who consumed 8 fluid ounces/day of vegetable juice (p = 0.002 and < 0.001 for LSD and Tukey HSD, respectively); the difference between the 8 fluid ounce/day and 16 fluid ounce/day juice groups was non-significant (p = 0.40 and 0.68 for LSD and Tukey HSD, respectively).

Given study attrition, the adjusted and aggregated models were computed. These data showed significant differences over time between the aggregated groups consuming juice compared to the control group for carbohydrates (F = 3.3, p = 0.05), total sugars (F = 3.3, p = 0.05; data not shown), vitamin C (F = 4.6, p = 0.02), and potassium (F = 3.9, p = 0.03). Those consuming vegetable juice significantly reduced their carbohydrate and sugar intake over time compared to those not consuming juice. Additionally, those drinking juice significantly increased their potassium intake over time compared to those not drinking it. Participants consuming vegetable juice experienced large increases over time in vegetable intake relative to the no juice group (see Table 5) using MyPyramid (F = 3.6, p = 0.04) vegetable categorization method. However, when vegetable juice intake is excluded from being counted in the dietary intake, there were no significant changes observed over time or group by time interactions with regard to usual vegetable intake.

### Metabolic Syndrome

Based on group assignment, there was a significant difference in the percent of subjects who met the metabolic syndrome criterion of elevated triglycerides at the end of the 12 week study, (Chi-square = 7.9; p = 0.02) with 40.0% still meeting the criterion in the no juice control group, while 5.6% and 13.6% met the criterion in the 8 fluid ounce/day and 16 fluid ounce/day juice groups respectively. A follow-up logistic model with the juice groups aggregated (comparing any vegetable juice consumption to none) and adjusting for age, education, and gender was developed to predict meeting the elevated triglyceride criterion at 12 weeks. This model demonstrated that those drinking the vegetable juice (10.0%) were less likely (OR = 0.91; p = 0.01) to meet the triglyceride criterion for metabolic syndrome than those not receiving the juice (40.0%). The overall model was statistically significant (Chi-square = 19.5; p = 0.003). There were no significant differences among groups with any of the other metabolic syndrome criteria.

## Discussion

Diets rich in vegetables and fruits have been shown to help individuals reach and achieve a healthy weight [5] and improve cardiovascular disease risk [7,40]. This positive result has been attributed to the fact that vegetables and fruit are typically low in calories and have been shown to increase satiation [6,41]. However, adopting and maintaining a healthy lifestyle that includes a diet rich in vegetables, fruits, lean meats and low fat dairy products, is problematic for many individuals [8]. The current study examined whether including an easily accessible, portable vegetable-based beverage as part of a calorie-controlled DASH diet could increase vegetable intake and improve clinical characteristics of the metabolic syndrome in a group of individuals with a mean age of 49.8 years, predominately female (73%), with 84% self-identified as African American, Mexican American or other minority. During the 12 week study, participants received two individual counseling sessions with registered dietitians (at baseline and 6 weeks). Although everyone was counseled on a calorie-controlled DASH diet only those who were instructed to also incorporate 8 and 16 fluid ounces of vegetable juice per day significantly increased their vegetable consumption and significantly reduced their carbohydrate intake. Regardless of whether vegetable servings included or excluded the "starchy vegetables" (such as those defined by the Diabetic Vegetable Exchanges [42]), subjects consumed significantly more vegetables in the juice treatment groups.

This dietary practice translated to a significant amount of weight loss in the vegetable juice groups compared to those who did not incorporate the vegetable juice into the DASH diet. The amount of weight lost was modest, approximately 0.33 lb per week. But, this positive, "small-step" change is thought to be successful [43] and in our intervention resulted in a significantly greater weight loss over the 12 weeks in the group that incorporated vegetable juice into the DASH diet compared to the group that did not drink the juice.

Individuals with metabolic syndrome are at higher risk for both diabetes and atherosclerotic cardiovascular disease. Weight reduction is known to improve risk factors associated with metabolic syndrome [44,45]. In the current study, after 12 weeks of vegetable juice consumption, the modest reduction in weight (<5% for most subjects), translated to a lower percentage of subjects that met the metabolic syndrome criteria for high triglycerides (i.e. >150 mg/dl). No significant changes were observed in any of the other measured metabolic or cardiovascular risk factors. However, we did observe a significant decrease in plasma leptin. Leptin is synthesized and secreted from adipocytes and is highly correlated with energy storage in adipose tissue [46,47]. Analogous to our results, the observation that changes in leptin and triglycerides parallel weight loss, regardless of mechanism to reduce weight, has been observed by other investigators [48,49].

It is important to note that the DASH diet instructions emphasized including vegetables of all forms in their daily diets, but only those groups provided with the simple intervention of adding vegetable juice significantly increasing vegetable intake. According to subjects' responses on the Beverage Consumption questionnaires, the juice was an acceptable addition to their diets. Apart from the vegetable juice, our subjects would not have met vegetable recommendations. Many different population groups do not meet current vegetable recommendations [50-52]. Inadequate vegetable intake is a widespread issue [53]. Although campaigns promoting vegetables and fruits, such as the 5-A-Day program, have been publicized in the media and the public recognizes them [54], there is disconnect between the recommendations and typical consumption [6,50,51,55]. In agreement with the above, in the current study, despite our DASH diet education, including an emphasis on vegetable intake, we observed no increases in dietary vegetables, apart from the added vegetable juice over time.

Education alone typically does not seem to relate to significant dietary improvements. McGee et al. studied a population with similar education to the present study, with approximately half of the participants with a high school education or less, and found that barriers to change towards a more healthful diet included lack of knowledge and skills [9]. Despite the fact that we provided our subjects with DASH diet knowledge and food preparation tips, our participants still did not meet their vegetable recommendations unless a vegetable juice beverage was provided to them. Although a serious disease may motivate changes in dietary behavior, our subjects had cardiovascular risk factors, rather than a major cardiovascular event, which may have reduced their incentive to follow DASH diet guidelines [9]. For example, Campbell et al. found that in a predominantly female population, consisting of a high percentage of minorities, it was possible to increase knowledge of infant feeding through education but that knowledge alone did not elicit change in dietary behaviors [56]. Dietary interventions and education targeting minorities is especially difficult [57,58]. However, our study showed beneficial dietary changes in minorities. Participants provided a vegetable beverage greatly enhanced their vegetable consumption, something the DASH counseling and materials alone, were unable to achieve. Consistent with the current study, Weerts et al. [59] reported that African American women, given nutritional and behavioral education, were more likely to increase their consumption of vegetables and consequently lose weight when they were provided with gift cards that were explicitly for vegetables and fruits.

While studies have observed blood pressure reductions in trials incorporating tomato based products [21,22], we did not. These studies used tomato-based extracts, rather than a tomato-based juice. In addition to a lack of effect on blood pressure, vegetable juice consumption also did not correlate to an improvement in oxidative stress parameters, although a significant increase in vitamin C intake was observed in the vegetable juice groups. We note that other markers of antioxidant and oxidative stress may have yielded different results [60].

Limitations of the current study design include its short duration of 12 weeks. A longer study could provide data on weight loss maintenance, a key factor for weight control and health. In addition, since attrition was slightly higher than anticipated, even though it was not significantly different among groups, it became necessary to aggregate the groups consuming the vegetable juice for statistical power. While examination of the variables using the LOCF and ITT models did not reach statistical significance, they do show the same basic trends as the aggregated models. When looking at the more conservative models combined with the aggregated model, we acknowledge that our findings are preliminary and more research is needed. Another limitation to our study was the relatively modest-to-low rate of adherence among the 16 fluid ounces/day group. There were no significant differences in the adjusted models for weight loss among all three groups, however, on average, those who consumed 16 fluid ounces/day lost less weight than those who consumed 8 fluid ounces/day. It is difficult to know why the 16 fluid ounces/day group may not have been as effective as the 8 fluid ounces/day group in terms of weight loss. One possibility is the relatively low adherence to the intervention protocol in those assigned to consume a greater volume of juice. This finding indicates that it may be difficult, in a clinical or public health setting to recommend drinking 16 fluid ounces/day of vegetable juice.

## Conclusion

In conclusion our study demonstrates that the incorporation of vegetable juice is a simple and effective way to help meet vegetable recommendations and improve Vitamin C and potassium intake. Data from this study also suggest the potential of using a low sodium vegetable juice in conjunction with a calorie restricted diet to aid in weight loss in overweight individuals with metabolic syndrome.

## Competing interests

This work was supported by resources from the Campbell Soup Company. CS Khoo and BL Winters are employees of Campbell Soup Company and hold stock there. CL Keen and JP Foreyt are members of Campbell Soup Company's Vegetable Plant Advisory Panel. SF Shenoy, WSC Poston, RS Reeves, AG Kazaks, RR Holt, HJ Chen, CK Haddock do not have any financial interests to declare.

## Authors' contributions

SFS interpreted data, drafted and critically reviewed the manuscript, and gave final approval of the version to be published. WSCP analyzed and interpreted data, drafted and critically reviewed manuscript and gave final approval of the version to be published. RSR acquired and interpreted data, drafted and critically reviewed manuscript, and gave final approval of the version to be published. AGK interpreted data, drafted and critically reviewed the manuscript, and gave final approval of the version to be published. RRH made substantial contributions to conception and design of study, acquired and interpreted data, drafted and critically reviewed the manuscript, and gave final approval of the version to be published. CLK made substantial contributions to conception and design of study, interpreted data, drafted and critically reviewed the manuscript, and gave final approval of the version to be published. HJC acquired data, drafted and critically reviewed the manuscript, and gave final approval of the version to be published. CKH analyzed and interpreted data, drafted and critically reviewed manuscript and gave final approval of the version to be published. BLW made substantial contributions to conception and design of study, interpreted data, drafted and critically reviewed the manuscript, and gave final approval of the version to be published. CSK made substantial contributions to conception and design of study, interpreted data, drafted and critically reviewed the manuscript, and gave final approval of the version to be published. JPF made substantial contributions to conception and design of study, interpreted data, drafted and critically reviewed the manuscript, and gave final approval of the version to be published.
